# High neutrophil‐to‐lymphocyte ratio as an early sign of cardiotoxicity in breast cancer patients treated with anthracycline

**DOI:** 10.1002/clc.23966

**Published:** 2023-01-20

**Authors:** Ranny Baruch, David Zahler, Lior Zornitzki, Yaron Arbel, Zach Rozenbaum, Joshua H. Arnold, Ari Raphael, Shafik Khoury, Shmuel Banai, Yan Topilsky, Livia Kapusta, Michal Laufer‐Perl

**Affiliations:** ^1^ The B. Rappaport Faculty of Medicine, Technion Haifa Israel; ^2^ Department of Cardiology Sackler School of Medicine, Tel Aviv University Tel Aviv Israel; ^3^ Department of Internal Medicine B, Sackler School of Medicine Tel Aviv University Tel Aviv Israel; ^4^ Sackler School of Medicine Tel Aviv University Tel Aviv Israel; ^5^ Department of Medicine University of Illinois at Chicago Chicago Illinois USA; ^6^ Oncology Department, Sackler School of Medicine Tel Aviv University Tel Aviv Israel; ^7^ Pediatric Cardiology Unit Tel‐Aviv Sourasky Medical Center Tel Aviv Israel; ^8^ Department of Pediatric Cardiology, Amalia Children's Hospital Radboud University Medical Centre Nijmegen The Netherlands

**Keywords:** cardio‐oncology, cardiotoxicity, echocardiography, NLR, strain

## Abstract

**Background:**

Cardiotoxicity, defined mainly as left ventricle (LV) dysfunction, is a significant side effect of anthracyclines (ANT) therapy. The need for an early simple marker to identify patients at risk is crucial. A high neutrophil‐to‐lymphocyte ratio (NLR) has been associated with poor prognosis in cancer patients; however, its role as a predictor for cardiotoxicity development is unknown.

**Objective:**

Evaluating whether elevated NLR, during ANT exposure, plays a predictive role in the development of cardiotoxicity as defined by LV global longitudinal strain (LV GLS) relative reduction (≥10%).

**Methods and Results:**

Data were prospectively collected as part of the Israel Cardio‐Oncology Registry. A total of 74 female patients with breast cancer, scheduled for ANT therapy were included. NLR levels were assessed at baseline (T1) and during ANT therapy (T2). All patients underwent serial echocardiography at baseline (T1) and after the completion of ANT therapy (T3). NLR ≥ 2.58 at T2 was found to be the optimal predictive cutoff for LV GLS deterioration. A relative LV GLS reduction ≥10% was significantly more common among patients with high NLR (50% vs. 20%, *p* = .009). NLR ≥ 2.58 at T2 increases the risk for LV GLS reduction by fourfold (odds ratio [OR]: 4.63, 95% confidence interval [CI]: 1.29–16.5, *p* = .02), with each increase of 1‐point NLR adding an additional 15% risk (OR: 1.15, 95% CI: 1.01–1.32, *p* = .046).

**Conclusions:**

Our study provides novel data that high NLR levels, during ANT exposure, have an independent association with the development of LV dysfunction. Routine surveillance of NLR may be an effective means of risk‐stratifying.

## 1 INTRODUCTION

Breast cancer is the most prevalent type of cancer in women.^1^ While advancements in cancer therapy have led to a significant reduction in morbidity and mortality, the high burden of short‐ and long‐term side effects brought about by modern medical therapy plays a significant role in patient outcomes.^2^, ^3^ Cardiotoxicity is the most significant complication and is manifested mainly as left ventricle (LV) dysfunction.^4^, ^5^ Anthracyclines (ANT), in particular doxorubicin, remains a cornerstone in chemotherapy regimens for the treatment of patients diagnosed with breast cancer,^5^ and its cardiotoxic effects are characterized by dose‐dependent irreversible LV dysfunction.^6^, ^7^ Early detection of ANT‐related cardiac injury and clinician intervention is, therefore, paramount for the prevention of the development or worsening of LV dysfunction and heart failure (HF) in this patient population.^8^ Currently, two‐dimensional speckle‐tracking echocardiography and the use of LV global longitudinal strain (LV GLS) is considered the optimal parameter for early detection of subclinical LV dysfunction in cancer patients.^9^, ^10^, ^11^, ^12^

The absolute neutrophil‐to‐lymphocyte ratio (NLR), a simple measure using basic cell counts, has been found to be positively associated with increased mortality in cancer patients as its number increases.^13^, ^14^ This has been suggested to be due to the fact that neutrophils are proinflammatory and can contribute to the progression of cancer, while lymphocytes have been found to act as tumor inhibitors^15^ and an elevated NLR in cancer patients has been associated with poor prognosis.^14^, ^16^ Similarly, a number of studies have shown that elevated NLR in patients presenting with myocardial infarction is related to a major adverse cardiac event (MACE), as well as all‐cause mortality,^17^, ^18^ further suggests a relation between an immune reaction and cardiovascular disease (CVD) outcomes. However, data evaluating the role of NLR in predicting cardiotoxicity development in cancer patients is scarce.

The aim of our study was to evaluate whether elevated NLR, in patients diagnosed with breast cancer and treated with ANT, plays a predictive role in the development of cardiotoxicity, as defined by LV GLS reduction.

## 2 METHODS

### 2.1 Study population

The study population is part of the Israel Cardio‐Oncology Registry (ICOR) —a prospective registry enrolling patients evaluated in the cardio‐oncology clinic at the Tel Aviv Sourasky Medical Center. All patients signed informed consent at the first clinic visit. The registry was approved by the local ethics Tel Aviv Sourasky committee (Identifier: 0228‐16‐TLV) and is registered on clinicaltrials.gov (Identifier: NCT02818517). Included in the cohort were female patients diagnosed with breast cancer and scheduled for doxorubicin therapy with a cumulative dose of doxorubicin ≥180 mg/m^2^. All patients performed at least two echocardiography evaluations; at baseline, before doxorubicin exposure (T1), and at the end of doxorubicin therapy (T3). The majority of the patients performed additional echocardiography evaluation following the third ANT cycle (T2). Blood samples, including NLR, were taken at baseline (T1) and during doxorubicin exposure (T2), in close proximity to the echocardiography exam (T2). Exclusion criteria included age below 18, male gender, reduced baseline LV function (LV ejection fraction [LVEF] < 53%), a history of cardiac disease (myocardial infarction, myocarditis, severe valvular diseases), past ANT therapy, dexrazoxane (cardioxane) therapy and no documentation of NLR values or LV GLS assessment.

### 2.2 Study protocol

A complete baseline medical history, cardiac risk factors, medical treatment, and blood tests were noted in all patients from the electronic medical charts. All patients were treated with an AC protocol (intravenous [IV] doxorubicin 60 mg/m^2^ + IV cyclophosphamide 600 mg/m^2^ every 2‐3 weeks for 4 cycles), following in most cases, IV paclitaxel 80 mg/m^2^ every week for 12 cycles. Patients presenting with a positive humanized anti‐human epidermal growth factor receptor 2 (HER2) were treated with IV trastuzumab (a humanized anti‐HER2 monoclonal antibody) 8 mg/kg loading dose/6 mg maintenance and then IV pertuzumab (a humanized anti‐HER2 monoclonal antibody) 840 mg loading dose/420 mg maintenance. The number of cycles for trastuzumab and pertuzumab was determined by the treating physician.

**Figure 1:**
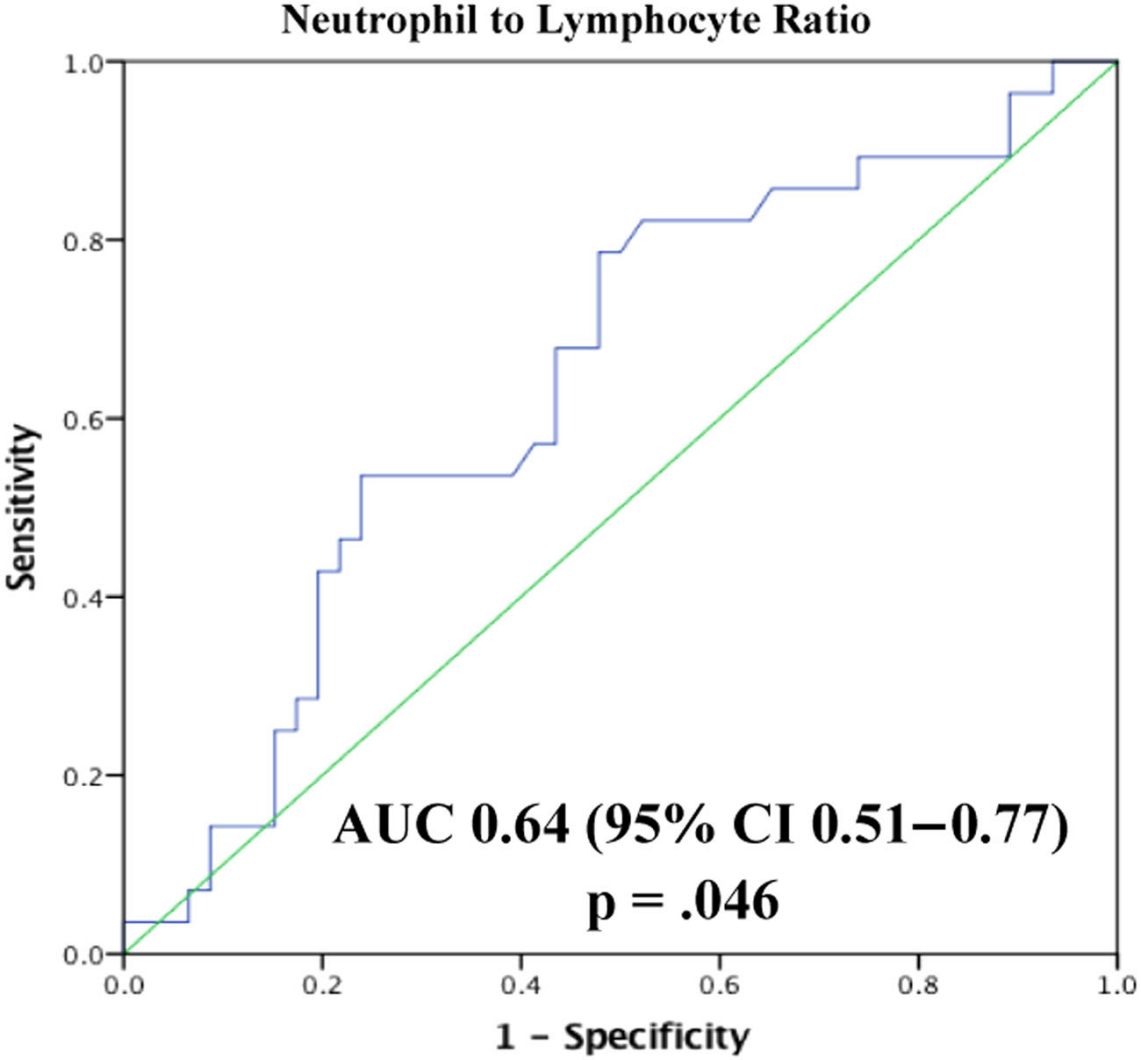
Receiver operator characteristic curve analysis of neutrophil‐to‐lymphocyte ratio for prediction of left ventricle global longitudinal strain reduction. AUC, area under the curve; CI, confidence interval.

All echocardiography exams evaluated systolic and diastolic function, including LV GLS, as described in the echocardiography section. NLR levels, measured as the ratio between absolute neutrophil count to absolute lymphocyte count, were evaluated at baseline, before doxorubicin exposure (T1), and during doxorubicin exposure (T2), before echocardiography follow‐up at the end of doxorubicin therapy (T3). The study population was stratified into two groups based on NLR values, according to the optimal predictive cutoff for LV GLS deterioration at T3, determined by a receiver operator characteristic (ROC) curve (Figure 1).

A significant reduction in LV GLS from T1 to T3 was defined as a relative reduction of ≥10%, as accepted by previous studies.^19^

### 2.3 Echocardiography

Follow‐up echocardiographic examinations were performed using the same strict protocol, personnel, and equipment (General Electric system, model Vivid S70). Routine LV echocardiographic parameters included LV diameters and LVEF.^20^ Early transmitral flow velocity (E), late atrial contraction (A) velocity, and early diastolic mitral annular velocity (septal and lateral e′) were measured in the apical four‐chamber view to provide an estimate of LV diastolic function.^20^ The peak E/peak e′ (E/e′) ratio was calculated (septal, lateral, and average mitral E/e′ ratio), and the deceleration time of the E wave was measured. The left atrium volume index was calculated using the biplane area length method at end‐systole. Images were acquired using a high frame rate (>50 frames/s),^21^ and thereafter stored digitally for offline analysis. LV GLS was measured using EchoPac STE software and tracking within an approximately 5 mm wide region of interest. An end‐systolic frame was used to initialize LV boundaries which were then automatically tracked throughout the cardiac cycle. Manual corrections were performed to optimize boundary tracking as needed. Optimization of images for endocardial visualization through adjustment of gain, compress, and time‐gain compensation controls were done before acquisition.

### 2.4 Statistical analysis

Categorical variables were expressed as frequency and percentages. *χ* ^2^ test was used to evaluate the association between these variables. The distribution of continuous variables was assessed using the Kolmogorov–Smirnov test. Normally distributed continuous variables were described as mean and standard deviation, these variables were compared using the independent samples *t‐test*. Non‐normally distributed continuous variables were expressed as the median and interquartile range (IQR), Mann–Whitney *U* test was used for comparison. Changes in continuous laboratory variables over time were assessed with paired‐samples *t‐test* if normally distributed and Wilcoxon signed‐rank test if not normally distributed. ROC curve analysis was performed to identify cutoff points of NLR (at which sensitivity and specificity would be optimal) for the prediction of future LV GLS reduction. Areas under the curves (AUC) were calculated as measures of the accuracy of the test. The influence of NLR on the risk for LV GLS reduction was evaluated using multivariate binary logistic regression. Variables included were those showing a significant predictive value in univariate analysis (hypertension[HTN]), and variables with a known effect on LV GLS (age, BSA, DM, trastuzumab, etc.). Adjusted odds ratio (OR) with a 95% confidence interval (CI) was reported for all variables. A two‐tailed *p* < .05 was considered significant for all analyses. All analyses were performed with the IBM SPSS 23.0 software (SPSS Inc.).

## 3 RESULTS

### 3.1 Study population

From July 2016 to March 2021, 143 patients were identified; of which, 74 met the inclusion criteria. Excluded were 23 patients due to lack of documentation of NLR values, 45 patients due to lack of LV GLS measurements before or following the completion of doxorubicin therapy, and 1 due to male gender.

### 3.2 ROC and AUC

Evaluating NLR at T1, both ROC curve analysis (AUC: 0.59, 95% CI: 0.44–0.75, *p* = .24) and binary logistic regression (OR: 1.07 [95% CI: 0.81–1.4], *p* = .65] were nonsignificant for predicting future LV GLS deterioration at T3. ROC curve analysis with NLR at T2 for predicting future LV GLS deterioration at T3 resulted in an AUC of 0.64, 95% CI: 0.51–0.77, *p* = .046 (Figure 1). According to this analysis, the optimal cutoff value was ≥2.58, with 79% sensitivity and 52% specificity. Overall, 44 (59%) patients presebted with NLR ≥ 2.58 at T2.

### 3.3 Baseline parameters

All patients were female with a mean age of 50 ± 12 years. None of the patients had a history of coronary artery disease. Cardiac risk factors were not frequent and ranged from 7% to 22% (Table 1). A total of 18% of patients were treated with cardioprotective therapies, such as angiotensin‐converting enzyme inhibitors (ACE‐I), angiotensin II receptor blockers (ARB), or beta‐blockers, mainly indicated for HTN. At T3, almost all patients (99%) completed four cycles of doxorubicin therapy (cumulative dose of 240 mg/m^2^). Sixteen (22%) patients were treated with trastuzumab, 14 (19%) with pertuzumab, and 16 (22%) patients had undergone chest radiations. All therapies were initiated following the completion of ANT therapy. Patients with low NLR values were more likely to have HTN (30% vs 11%, *p* = .04) without significant differences in other clinical and therapeutic aspects, as stated in Table 1.

**Table 1:** Baseline characteristics

	Total (*n* = 74)	NLR < 2.58 at T2 (*n* = 30)	NLR ≥ 2.58 at T2 (*n* = 44)	*p* Value
Age (years), mean ± SD	50 ± 12	53 ± 12	49 ± 11	.18
BSA (m^2^), median (IQR)	1.69 (1.59–1.84)	1.71 (1.60–1.79)	1.69 (1.57–1.90)	.92
*Medical history*				
Hypertension, *n* (%)	14 (18.9)	9 (30.0)	5 (11.4)	**.04**
Diabetes mellitus, *n* (%)	5 (6.8)	4 (13.3)	1 (2.3)	.15
Hypercholesterolemia, *n* (%)	9 (12.2)	5 (16.7)	4 (9.1)	.32
Smoking (current or past), *n* (%)	16 (21.6)	4 (13.3)	12 (27.3)	.15
*Concomitant anticancer therapy*				
Paclitaxel, *n* (%)	71 (96)	27 (90)	44 (100)	.06
Carboplatin, *n* (%)	10 (14)	3 (10)	7 (16)	.46
Trastuzumab, *n* (%)	16 (22)	5 (17)	11 (25)	.39
Left chest radiation, *n* (%)	32 (43)	11 (37)	21 (48)	.35
*Medications*				
Beta‐blockers, *n* (%)	3 (4.1)	2 (6.7)	1 (2.3)	.56
ACEI/ARB, *n* (%)	13 (17.6)	7 (23.3)	6 (13.6)	.28
ACEI/ARB/beta‐blockers, *n* (%)	13 (17.6)	7 (23.3)	6 (13.6)	.28
Statins, *n* (%)	10 (13.5)	5 (16.7)	5 (11.4)	.51
Antiaggregation, *n* (%)	5 (6.8)	1 (3.3)	4 (9.1)	.64
*Laboratory data at T1*				
Hemoglobin (g/dl), mean ± SD	12.9 ± 1.0	12.8 ± 1.0	12.9 ± 1.0	.61
White blood cell count (10^9^/L), mean ± SD	6.7 ± 2.4	6.3 ± 2.1	7.1 ± 2.5	.61
Platelet count (10^9^/L), median (IQR)	257 (209–297)	259(215–305)	251(201–297)	.42
Creatinine (mg/dl), mean ± SD	0.68 ± 0.15	0.69 ± 0.12	0.66 ± 0.15	.31
*Laboratory data at T2*				
Hemoglobin (g/dl), mean ± SD	11.3 ± 1.2	11.2 ± 1.2	11.4 ± 1.2	.58
White blood cell count (10^9^/L), median (IQR)	5.9 (3.8–7.9)	4.0 (2.9–5.9)	7.1 (5.4–9.5)	**<.001**
Platelet count (10^9^/L), median (IQR)	226 (186–298)	258 (184–324)	221 (190–282)	.37
Creatinine (mg/dl), mean ± SD	0.64 ± 0.17	0.65 ± 0.15	0.63 ± 0.17	.67

*Note*: Bold values are significant *p* < .05.

Abbreviations: ACEI, angiotensin‐converting enzyme inhibitors; ARB, angiotensin II receptor blockers; BB, beta‐blockers; BSA, body surface area; IQR, interquartile range; NLR, neutrophil‐to‐lymphocyte ratio; SD, standard deviation; T1, baseline before doxorubicin therapy; T2, during doxorubicin exposure.

### 3.4 Laboratory parameters

**Figure 2:**
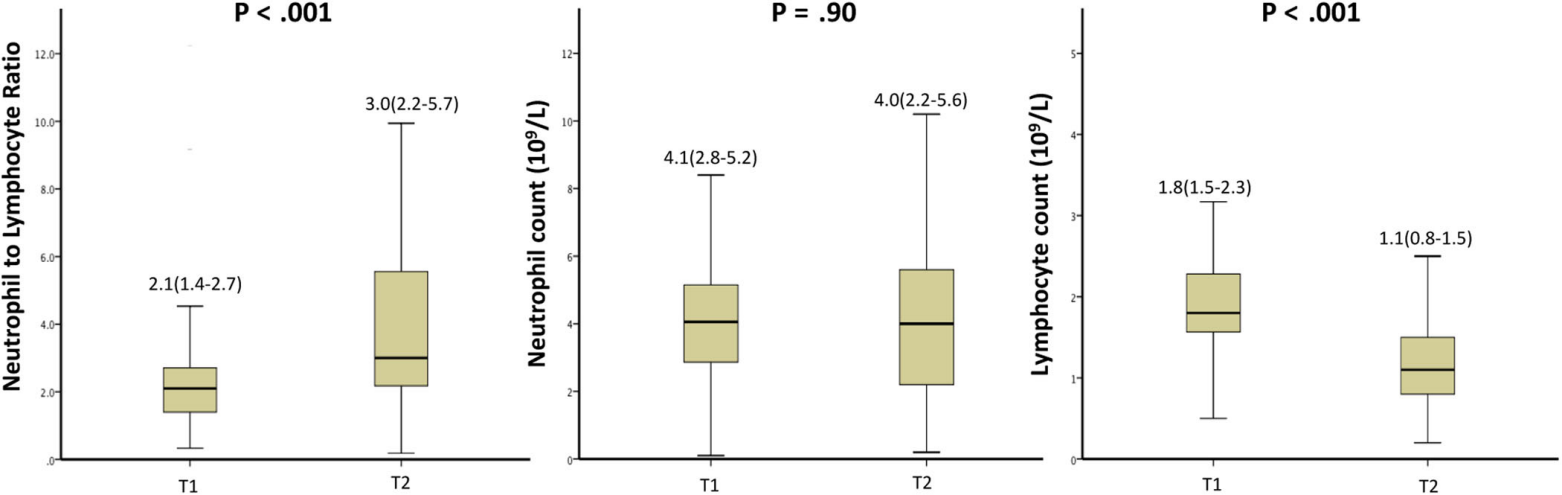
Changes in neutrophil‐to‐lymphocyte ratio values from Timepoint 1 (before doxorubicin therapy) to 2 (during doxorubicin therapy)

The median time elapsed from the first doxorubicin treatment to NLR evaluation at T2 was 44 (IQR: 28–64) days, which in the majority of the patients was following the third cycle of ANT with a cumulative dose of ≥180 mg/m^2^. A significant increase from T1 to T2 was observed for NLR values (T1 2.1 [1.4–2.7] vs. 3.0 [2.2–5.7], *p* < .001]. This change was mainly driven by a reduction in absolute lymphocyte counts (T1 1.8 [1.5–2.3] vs. T2 1.1 [0.8–1.5], *p* < .001), while absolute neutrophil counts did not differ significantly between both blood samples (T1 4.1 [2.8–5.2] vs. T2 4.0 [2.2–5.6], *p* = .90) (Figure 2). As displayed in Table 1, no difference was observed between study groups for hemoglobin or platelet levels, neither in T1 nor T2. White blood cell counts were significantly higher in the high NLR group only in T2 (*p* < .001). Serum creatinine levels were similar between both groups at both time points.

### 3.5 Outcomes

As displayed in Table 2, baseline echocardiographic examination (T1) showed no difference in LVEF (*p* = .31), LV GLS (*p* = .58), tricuspid annular plane systolic excursion (*p* = .62), or diastolic dysfunction ≥ Grade 2 (*p* = .16).

**Table 2:** Echocardiography parameters

	Total (*n* = 74)	NLR < 2.58 at T2 (*n* = 30)	NLR ≥ 2.58 at T2 (*n* = 44)	*p* Value
*Baseline echocardiography at T1*				
LVEF (%), mean ± SD	**60 ± 1.2**	**59 ± 0.9**	**60 ± 1.3**	**.31**
LV GLS (%), mean ± SD	−21.61 to 1.95	−21.46 to 1.93	−21.72 ± 1.97	.58
LV GLS > (−18)%, *n* (%)	2 (2.7)	0 (0)	2 (4.5)	.51
LVEDD (mm), median (IQR)	44 (42–46)	43 (41–45)	45 (42–47)	.08
IVS (mm), median (IQR)	9 (8–10)	9 (8–11)	9 (8–10)	.12
LAVI (ml/m^2^), median (IQR)	27 (22–33)	26 (22–35)	27 (23–33)	.62
TAPSE (mm), mean ± SD	24.8 ± 3.5	25.0 ± 3.6	24.6 ± 3.5	.62
Diastolic dysfunction ≥ Grade 2, *n* (%)	2 (6.7)	2 (6.7)	0 (0)	.16
*LV GLS changes from T1 to T3*				
Absolute LV GLS reduction, mean ± SD	1.9 ± 1.9	1.3 ± 1.7	2.3 ± 2.1	**.03**
Relative LV GLS reduction (%), mean ± SD	8.7 ± 8.8	6.1 ± 6.9	10.6 ± 9.6	**.02**
LV GLS relative reduction ≥ 10%, *n* (%)	28 (38)	6 (20)	22 (50)	**.009**

*Note*: Bold values are significant *p* < .05.

Abbreviations: IQR, interquartile range; IVS, interventricular septum; LAVI, left atrial volume index; LVEDD, left ventricular end‐diastolic diameter; LVEF, left ventricle ejection fraction; LV GLS, left ventricular global longitudinal strain; NLR, neutrophil‐to‐lymphocyte ratio; SD, standard deviation; T1, baseline before doxorubicin therapy; T2, during doxorubicin exposure; T3, at the end of doxorubicin therapy; TASPE, tricuspid annular plane systolic excursion.

The mean relative LV GLS reduction from T1 to T3 differed significantly between patients with NLR at T2 ≥ 2.58 and patients with NLR < 2.58 (10.6 ± 9.6 vs. 6.1 ± 6.9, *p* = .02, respectively; Table 2). Patients with NLR ≥ 2.58 at T2 were more than twice as likely to develop a relative LV GLS reduction ≥10% as compared to patients with NLR < 2.58 (50% vs. 20%, *p* = .009, respectively).

As presented in Table 3, in a multivariate binary logistic regression model, including baseline characteristics (age, body surface area), medical history (HTN, diabetes mellitus, hyperlipidemia, smoking status), therapeutic aspects (left breast radiation therapy, trastuzumab therapy), other blood count values (hemoglobin and platelets), and concomitant medication administration (ACE‐I, ARB, beta‐blockers), NLR ≥ 2.58 at T2 was shown to be a strong and independent predictor for future LV GLS deterioration with a fourfold increase in relative risk (OR: 4.63, 95% CI: 1.29–16.5, *p* = .02, Model 2). In a second model, each 1‐point rise in NLR value was associated with an additional 15% increased risk for LV GLS deterioration (OR: 1.15, 95% CI: 1.01–1.32, *p* = .046, Model 1). Besides NLR, only treatment with trastuzumab independently predicted LV GLS deterioration. Neither absolute neutrophil count alone (OR: 1.12, 95% CI: 0.98–1.29, 0.08) nor absolute lymphocyte count alone (OR: 1.02, 95 CI%: 0.35–2.97, *p* = .98) predicted LV GLS reduction in the same multivariate regression model.

**Table 3:** Multivariate binary regression models for prediction of LV GLS reduction

	Model 1		Model 2	
	OR (95% CI)	*p* Value	OR (95% CI)	*p* Value
NLR ≥ 2.58 at T2			4.63 (1.29–16.5)	**.02**
NLR (continuous)	1.15 (1.01–1.32)	**.046**		
Age (years)	0.95 (0.89–1.02)	.15	0.98 (0.92–1.04)	.43
BSA (m^2^)	3.32 (0.28–39.8)	.34	1.84 (0.15–22.9)	.63
Hypertension	2.04 (0.17–25.1)	.64	2.78 (0.22–35.5)	.43
Diabetes mellitus	0.45 (0.02–9.23)	.61	0.59 (0.03–11.3)	.73
Hypercholesterolemia	4.48 (0.36–55.8)	.24	2.54 (0.25–26.1)	.43
Smoking (current or past)	0.49 (0.11–2.15)	.35	0.60 (0.15–2.44)	.48
Left breast radiation therapy	1.26 (0.39–4.04)	.69	1.03 (0.32–3.32)	.96
Trastuzumab	4.90 (1.33–18.0)	**.02**	4.01 (1.09–14.7)	**.04**
Hemoglobin (g/dl)	0.92 (0.56–1.51)	.75	1.01 (0.61–1.65)	.98
Platelet count (10^9^/L)	1.002 (0.99–1.008)	.46	1.003 (0.99–1.008)	.39
ACEI/ARB/BB therapy	0.33 (0.02–6.22)	.46	0.39 (0.02–6.83)	.53

*Note*: Bold values are significant *p* < .05.

Abbreviations: ACEI, angiotensin‐converting enzyme inhibitors; ARB, angiotensin II receptor blockers; BB, beta‐blockers; BSA, body surface area; CI, confidence interval; LV GLS, left ventricle global longitudinal strain; NLR, neutrophil‐to‐lymphocyte ratio; OR, odds ratio.

Models 1 and 2 resulted in Nagelkerke *R* ^2^ values of 0.23 and 0.25, respectively. Hosmer–Lemeshow test was nonsignificant for both models (Model 1, *p* = .15 and Model 2, *p* = .92).

## 4 DISCUSSION

We evaluated, for the first time to our knowledge, the association between elevated NLR values and the development of cardiotoxicity among patients with breast cancer. We found that NLR ≥ 2.58, during ANT exposure (T2), was associated with a fourfold increased risk for developing significant LV GLS deterioration, with incremental increases of 1‐point NLR adding an additional 15% risk.

The use of NLR measurements as a surrogate marker of poor outcomes is gaining popularity, both in the field of cancer and CVD. Past studies have shown that high baseline NLR values are associated with poor overall survival in different types of cancer, including breast cancer.^13^, ^14^ This may be explained^14^, ^22^, ^23^ by the fact that neutrophils have been shown to promote cancer development while lymphocytes play a key role in the immune reaction against neoplasm development and proliferation. Furthermore, when associating NLR with CVD, higher NLR values have also been associated with worsening CV outcomes and mortality among patients with ST‐elevation myocardial infarction and HF.^18^, ^24^ Arbal et al.^18^ have similarly shown an association between high NLR values and reduced LVEF. Relative neutrophilia reflects a systemic inflammatory physiologic state and promotes the creation of oxygen‐free radicals, thus perpetuating further myocardial tissue damage. Furthermore, associated lymphopenia, promoted by elevated cortisol levels during a physiologically stressed state, has been shown to promote apoptosis.^18^ While the role of high NLR as an independent predictor for poorer outcomes in both cancer and CVD patient populations has been well studied, its role in predicting cardiotoxicity development in cancer patients is yet unknown. Our study showed that high NLR, during ANT therapy (T2) was significantly associated with LV GLS deterioration, while baseline NLR (T1) was not, which is likely explained by the significant NLR increase from T1 to T2 (Figure 2) following ANT exposure. Of note, patients were also treated with granulocytes‐colony stimulating factor (GCSF) during ANT therapy which may have caused an increase in NLR. However, we observed that the change in NLR in our study was mainly a result of a reduction in the absolute lymphocyte count rather than an elevated absolute neutrophil count that is seen with GCSF exposure. Furthermore, based on the timing of the NLR increase, it is suggested that it was more likely a result of ANT exposure rather than a baseline inflammatory state from cancer. Importantly, there were no differences in baseline clinical characteristics or blood tests between the groups that may confound or falsely elevate the measured NLR or act as a risk predictor of LV GLS deterioration.

Our findings are consistent with recent papers, showing a statistically significant increase in NLR values over time in patients who developed immune checkpoint inhibitors‐associated myocarditis,^25^ as well as an^25^ association with MACE development.

In our study, the optimal NLR predictive cutoff value for LV dysfunction was found to be ≥2.58, in concordance with past studies of breast cancer patients that have used an NLR cutoff value of 3 as predictive for overall survival.^13^

With a growing need for improved risk stratification of patients at risk for the development of cardiotoxicity, the search for an early, easily accessible marker is necessary. Surveillance with serial LVEF measurements is advised for patients treated with ANT; however, LVEF reduction is usually evident only after significant, oftentimes irreversible, myocardial damage has occurred.^26^ NLR is an inexpensive, readily available marker that is performed routinely in a simple blood test, and thus has great potential utility as an early detection tool for cardiotoxicity.

Our study has several limitations. First, it is a single‐center study, however, its strength is the prospective nature that enrolls a homogenous population and contains uniformity of all echocardiography exams performed by the same vendor, technician, and interpreting physician. Second, we acknowledge that the relatively small number of patients reduces the statistical power of our results, and larger trials are needed. Third, the relatively short period of follow‐up did not allow us to evaluate the association or predictability between high NLR values and the development of LVEF reduction, cardiac morbidity, and all‐cause mortality.

## 5 CONCLUSIONS

In summary, our study provides novel data that high NLR, during ANT exposure, has an independent association with the development of LV GLS deterioration, a parameter of LV dysfunction. Routine surveillance of NLR values may be an effective and rapid means of risk‐stratifying and predicting early systolic dysfunction among patients with breast cancer following ANT therapy.

## CONFLICT OF INTEREST

The authors declare no conflict of interest.

## Data Availability

The datasets generated during and/or analyzed during the current study are not publicly available due to privacy hospital policy but are available from the corresponding author on reasonable request
